# *Ficus umbellata* Vahl. (Moraceae) Stem Bark Extracts Exert Antitumor Activities In Vitro and In Vivo

**DOI:** 10.3390/ijms18061073

**Published:** 2017-05-23

**Authors:** Kevine Kamga Silihe, Stéphane Zingue, Evelyn Winter, Charline Florence Awounfack, Anupam Bishayee, Nishil N. Desai, Leônidas João Mello, Thomas Michel, Francine Nzufo Tankeu, Derek Tantoh Ndinteh, Sara Honorine Riwom, Dieudonné Njamen, Tânia Beatriz Creczynski-Pasa

**Affiliations:** 1 Department of Biochemistry, Faculty of Sciences, University of Yaounde 1, Yaounde 812, Cameroon; kamkevine@yahoo.com (K.K.S.); yerema2003@yahoo.fr (F.N.T.); sarariwom@yahoo.fr (S.H.R.); 2 Department of Life and Earth Sciences, Higher Teachers’ Training College, University of Maroua, Maroua 55, Cameroon; stephanezingue@gmail.com; 3 Department of Animal Biology and Physiology, Faculty of Science, University of Yaoundé 1, Yaounde 812, Cameroon; c.awounfack@yahoo.fr (C.F.A.); dnjamen@gmail.com (D.N.); 4 Department of Pharmaceutical Sciences and Department of Biochemistry, Federal University of Santa Catarina, Florianópolis 88040-900, Brazil; eve_winter@hotmail.com (E.W.); leonidasjmj@gmail.com (L.J.M.); 5 Department of Applied Chemistry, Faculty of Sciences, University of Johannesburg, Doornfontein 2028, South Africa; tantohtantoh@gmail.com; 6 Department of Pharmaceutical Sciences, College of Pharmacy, Larkin University, Miami, FL 33169, USA; ndesai@ularkin.org; 7 Institute of Chemistry of Nice, Faculty of Sciences, University Côte d’Azur, Nice F-06108 Nice Cedex 2, France; thomasmichel.pro@hotmail.fr

**Keywords:** *Ficus umbellata*, apoptosis, estrogen-dependent tumors, DMBA, cell invasion

## Abstract

A *Ficus umbellata* is used to treat cancer. The present work was therefore designed to assess antitumor potentials of *F. umbellata* extracts in nine different cell lines. Cell cycle, apoptosis, cell migration/invasion, levels of reactive oxygen species (ROS), mitochondrial membrane potential (MMP), caspases activities as well as Bcl-2 and Bcl-xL protein content were assessed in MDA-MB-231 cells. The 7,12-dimethylbenz(a)anthracene (DMBA)-induced carcinogenesis in rats were also used to investigate antitumor potential of *F. umbellata* extracts. The *F. umbellata* methanol extract exhibited a CC_50_ of 180 μg/mL in MDA-MB-231 cells after 24 h. It induced apoptosis in MCF-7 and MDA-MB-231 cells, while it did not alter their cell cycle phases. Further, it induced a decrease in MMP, an increase in ROS levels and caspases activities as well as a downregulation in Bcl-2 and Bcl-xL protein contents in MDA-MB-231 cells. In vivo, *F. umbellata* aqueous (200 mg/kg) and methanol (50 mg/kg) extracts significantly (*p* < 0.001) reduced ovarian tumor incidence (10%), total tumor burden (58% and 46%, respectively), average tumor weight (57.8% and 45.6%, respectively) as compared to DMBA control group. These results suggest antitumor potential of *F. umbellata* constituents possibly due to apoptosis induction mediated through ROS-dependent mitochondrial pathway.

## 1. Introduction

Cancer is a group of diseases characterized by the unregulated proliferation of abnormal cells that invade and disrupt surrounding tissues [1]. It is a source of significant morbidity worldwide, responsible for over 8.2 million deaths per year and a serious public health problem in both developed and developing countries [2]. Breast and gynecological cancers (e.g., ovarian and uterus) are the major cause of cancer death in women of developing countries, and represent 19% of cancers worldwide [3]. According to the World Health Organization, the incidence of breast and gynecological cancers is predicted to increase in the future and 70% new cases will be observed in developing countries [2]. In Africa, the most common cancers in women are breast and cervical ones [4]. However, endometrial and ovarian cancers are also routinely observed. The incidence of ovarian cancer increases worldwide by about 10-fold in women during the peri- to post-menopausal period, when compared to premenopausal women [5]. With 3500 deaths per year, the ovarian cancer is the most lethal of gynecologic malignancies and a relatively uncommon cancer, making testing of drugs for primary prevention difficult [6,7].

Nowadays, therapeutic regimens against breast and gynecological cancers include radiotherapy, chemotherapy, immunotherapy and hormonal therapies, and, in advanced stages of the disease, surgery intervention can be required [8,9]. Despite substantial improvements in survival, the management of cancer is limited due to its high cost, scarcity of diagnosis and treatment, limited access to health services found in African countries [10], and most importantly the problem of resistance as well as high systemic toxicity of available chemotherapeutic drugs [11]. The need for primary prevention is worthwhile and novel efficacious, non-toxic and cost-effective therapeutic agents should be developed. Moreover, epidemiological and laboratory studies have provided evidence that naturally occurring dietary or plant components may exert protective effects against various cancers [12,13,14]. Increasingly, more medicinal plants are being screened for anticancer properties and may provide effective therapeutic agents.

The primary prevention of breast and gynecological cancers remains an important issue for developing countries, where 80% of the population still resorts to traditional medicine for their primary health care [15]. In this scope, *Ficus umbellata*, a plant found in subtropical regions, growing up to 6–10 m has been investigated. *F. umbellata* stem barks are used in Cameroonian traditional medicine system for the treatment of many gynecological conditions, such as amenorrhea, dysmenorrhea as well as for menopausal complaints [16]. In addition, it is reported that figs have been conventionally used for their therapeutic benefits as anticancer remedies [17,18]. Previous studies showed that *F. umbellata* extracts, which contain 7-methylcoumarin (Table 1) exhibited weak estrogenic effects in vitro and in vivo as the main bioactive constituent [19]. Since most of ethnopharmacological usages of *F. umbellata* have been recommended in the literature to be taken into consideration when selecting plants for anticancer screenings [12], the present work was designed to assess in vitro and in vivo antitumor potentials of this plant. In vitro, various cancer cells were exposed to *F. umbellata* aqueous (AE) and methanolic (MeOH) extracts, and the cytotoxicity, mechanism of cell death, cell cycle analysis, anti-migration and anti-invasion potential, reactive oxygen species (ROS) levels, mitochondrial transmembrane potential, caspase activities and Bcl-2 family proteins content were studied. In vivo, the chemopreventive effects of *F. umbellata* extracts were investigated against 7,12 dimethylbenz(a)anthracene (DMBA)-induced mammary and ovarian carcinogenesis in female Wistar rats.

## 2. Results

### 2.1. Preliminary Phytochemical Analysis

The phytochemical screening of *F. umbellata* AE and MeOH extracts showed that phenols, flavonoids, alkaloids, steroids, saponins, cardiac glycosides and tannin were present in both extracts. Their concentrations in total phenols, flavonoids, flavonols and alkaloids are depicted in Table 2. As observed in the table flavonoids were the most abundant secondary metabolites present in the dried AE and MeOH extracts. Flavonols were found in both extracts, while alkaloids were the less abundant. However, *F. umbellata* MeOH extract was found to contain more flavonoids, flavonols and alkaloids than AE extract.

### 2.2. Cytotoxicity of F. umbellata Extracts

**Figure 1:**
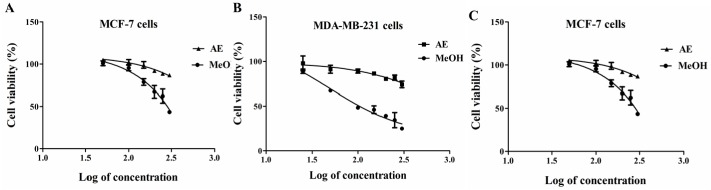
Concentration-dependent cytotoxicity of *F. umbellata* aqueous (AE) and methanolic extracts (MeOH) in breast cancer and non-tumoral cell lines. MCF-7 (**A**); MDA-MB-231 (**B**); and NIH-3T3 (**C**) cells were incubated with increasing concentrations (50–300 µg/mL) of extracts for 24 h and cell viability was evaluated by Alamar blue (resazurin) assay. The results are expressed in percentage as the mean ± SEM.

Figure 1 depicts the cytotoxicity response-curve of *F. umbellata* AE and MeOH extracts on breast cancer cells (MCF-7 and MDA-MB-231) and one non-tumoral cell line (NIH-3T3). As shown in the Figure, AE did not induce significant cytotoxicity up to 300 µg/mL, while the MeOH extract showed concentration-dependent cytotoxicity after 24 h of incubation and this effect was more pronounced in estrogen receptor-negative cells (MDA-MB-231, CC_50_ = 180 µg/mL) (Table 3A). Further, the 3 fractions from *F. umbellata* MeOH extract as well as 7-methoxycoumarin were assessed for their cytotoxicity in the same cell lines. Residue fraction (FU-R) and 7-methoxycoumarin (MC) did not showed cytotoxicity up to 300 µg/mL or 300 µM, respectively, in the tested cell lines, whereas dichloromethane fraction (FU-DCM) exhibited cytotoxicity at concentrations close to those of MeOH extract (Table 3A). To follow up, the cytotoxicity of *F. umbellata* MeOH extract and DCM fraction (FU-DCM) were assessed in five other cell lines (SF-295, 4T1, HUVEC, MCR-5, SK-MEL-28 and HCC 1995). Their CC_50_ were found to be quasi-similar in these cell lines (Table 3B), affording a selectivity index around two in human and one in murine cell lines (Table 3C). Since *F. umbellata* MeOH extract and FU-DCM fraction have the same cytotoxic activities, the understanding of underlying mechanisms of this plant was focused on MeOH extract.

### 2.3. Effects on Cell Death Mechanism

**Figure 2:**
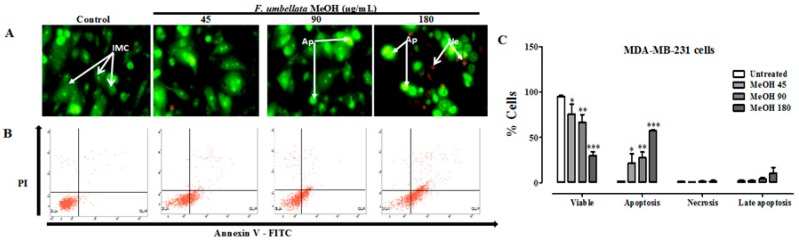
Effect of *F. umbellata* extracts on cancer cell death. Representative fluorescence microscopic images (400×) of cells double-stained with acridine orange (0.3 mg/mL) and ethidium bromide (1 mg/mL) (**A**); and dot plot representative of one experiment of apoptosis measurement by Annexin-V-FITC/PI staining (**B**). MDA-MB-231 cells were treated for 24 h to *F. umbellata* extracts at concentrations of 45, 90 and 180 µg/mL. (**C**) A graph showing the mean ± SEM of three independent experiments. * *p* < 0.05, ** *p* < 0.01 and *** *p* < 0.001 as compared with control. IMC, intact membrane cell; Ap, apoptotic cells; Ne, necrotic cells.

Apoptosis was evaluated by the qualitative acridine orange (AO)/ethidium bromide (EB) staining in this study. AO is absorbed by cells with intact or damaged membranes, and emits green fluorescence when it intercalates DNA. Ethidium bromide is taken only by cells with damaged membranes and emits red fluorescence fixed on DNA. Figure 2A and Figure S1A permit to distinguish three types of cells according to the fluorescence emission and the morphological aspect of chromatin condensation in the stained nuclei. Intact membrane cells (IMC) have uniform bright green nuclei with an organized structure. Apoptotic cells (Ap), which have green nuclei with condensed or fragmented chromatin and necrotic cells (Ne) have uniformly orange to red nuclei with an organized structure. It has been observed a chromatin condensation characteristic to apoptosis following *F. umbelleta* MeOH extract in MDA-MB-231 (Figure 2A) and MCF-7 (Figure S1A) cells lines.

Furthermore, the cell death mechanisms induced by F. umbellata MeOH extract were evaluated through flow cytometry. As depicted in dot plots (Figure 2B and Figure S1B), the viable cells had low FITC fluorescence and low PI fluorescence; early apoptotic cell showed high FITC fluorescence, but low PI fluorescence. The percentages of apoptotic and necrotic cells were 1.8% and 1.7%, respectively, in control cells. The concentration-dependent increase in the percentage of apoptotic cells with the maximum of 33.9% at 180 µg/mL of F. umbellata MeOH extract suggests that it induced apoptosis in the MDA-MB-231 (Figure 2C) and MCF-7 (Figure S1C) cells.

### 2.4. Effects on Cell Cycle

**Figure 3:**
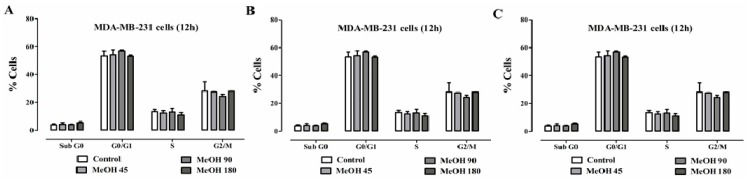
Effect of *F. umbellata* extracts on cell cycle distribution in MDA-MB-231 cells after different times of incubation: 12 h (**A**); 24 h (**B**); and 48 h (**C**). Cells were treated for 24 h with 45, 90 and 180 µg/mL of extracts and staining with PI. Following flow cytometry, cellular DNA profile was analyzed using the software WinMDI 2.9. Data represent the percentage of cell counts in each cell cycle phase. The results are expressed as the mean ± SEM of three independent experiments. No significant change was noted.

Flow cytometry was used to determine the effects of *F. umbellata* MeOH extract on the cell cycle progression of MCF-7 and MDA-MB-231 cells. It did not induce significant change in both MDA-MB-231 (Figure 3) and MCF-7 (Figure S2) cell cycle after 12, 24 and 48 h of treatment. Since effects of *F. umbellata* MeOH extract were the same in MDA-MB-231 and MCF-7 cells, we used MDA-MB-231 cells for evaluating anticancer effects of extract.

### 2.5. Effects on Cell Migration and Invasion

**Figure 4:**
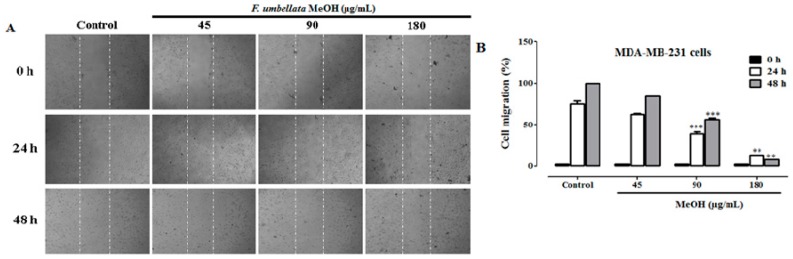
Effect of *F. umbellata* MeOH extract on cell migration potential. Microphotographs of one assay (**A**); and graphic representation of three independent wound-healing assay (**B**) in MDA-MB-231 cells migration after 24 and 48 h of treatment. ** *p* < 0.01, *** *p* < 0.001 as compared with control.

**Figure 5:**
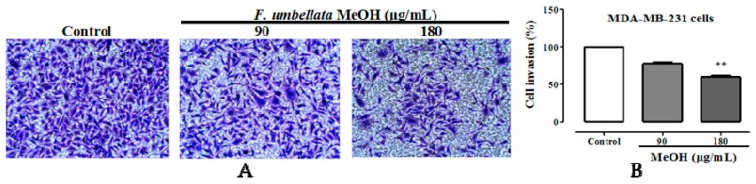
Microphotographs (100×) of violet crystal staining of MDA-MB-231 cells in transwell insert after 48 h of treatment (**A**); and graph of three independent assay (**B**). ** *p* < 0.01 compared to control.

It was observed a significant and concentration-dependent inhibition of MDA-MB-231 cells migration with *F. umbellata* MeOH extract at concentration of 90 and 180 µg/mL after 24 and 48 h of exposition (Figure 4). Further, we sought to determine the ability of *F. umbellata* MeOH extract to inhibit MDA-MB-231 cells invasion. It was observed a significant (*p* < 0.01) anti-invasion effect of MDA-MB-231 cells at the concentration of 180 µg/mL (Figure 5).

### 2.6. Effects on Mitochondrial Transmembrane Potential

**Figure 6:**
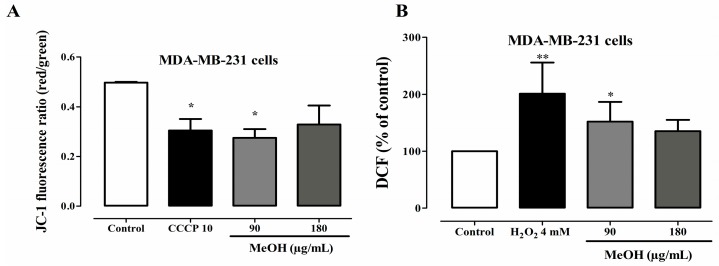
Effects of *F. umbellata* extract on oxidative stress parameters. Mitochondrial transmembrane potential (**A**); and intracellular reactive oxygen species (ROS) levels (**B**) in MDA-MB-231 cells treated with 90 and 180 µg/mL of *F. umbellata* MeOH extract for 24 h. The mitochondrial membrane potential was measured using the JC-1 fluorescent probe, while ROS level was determined using the DCHF-DA fluorescent probe by spectrofluorimeter. * *p* < 0.05 and ** *p* < 0.01 as compared to control. For Western blotting, β-actin was used as an internal control.

Since *F. umbellata* MeOH induced apoptosis, the next step was to investigate whether it acts by ROS-mediated mitochondrial dysfunction pathway. For this, a green fluorescent probe (JC-1) was used. The probe forms aggregates and emits red fluorescence peak at high mitochondrial membrane potential or forms monomers and emits a green fluorescence peak at low mitochondrial membrane potential. *F. umbellata* MeOH extract induced a reduction in mitochondrial transmembrane potential of MDA-MB-231 cells by reducing the red/green fluorescence ratio such as CCCP (10 µM) used as a positive control in this assay. However, statistical significance was reached only at a concentration of 90 µg/mL (Figure 6A).

### 2.7. Effects on ROS Levels

A fluorescence probe DCFH-DA was used for detecting the changes in the intracellular ROS level in this study. DCFH-DA is a fluorescent dye that diffuses through cell membranes and is hydrolyzed by intracellular esterases to DCFH. In the presence of ROS, DCFH is oxidized to DCF, which is fluorescent and its level corresponds to the level of generated ROS. An increase of ROS levels was observed in MDA-MB-231 cells following F. umbellata treatment. However, statistical significance was reached only at concentration of 90 µg/mL (Figure 6B).

### 2.8. Effects on Bcl-2 Family Protein Expression

**Figure 7:**
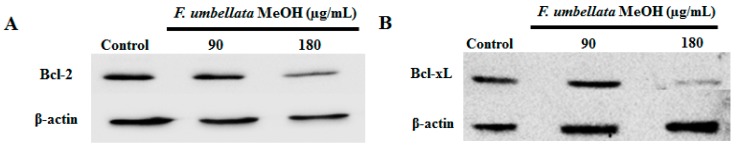
Effects of *F. umbellata* extract on Bcl2 and Bcl-xL content. Western blot analysis of Bcl-2 (**A**) and Bcl-xL (**B**) protein contents in MDA-MB-231 cells treated with 90 and 180 µg/mL of *F. umbellata* MeOH extract for 24 h.

Bcl-2 and Bcl-xL proteins are overexpressed in a variety of human cancers, and function as suppressors of apoptosis, resulting in the survival of malignant cells. The Bcl-2 and Bcl-xL antiapoptotic proteins content were measured using Western Blot in MDA-MB-231 cells. As depicted in Figure 7A,B, a reduction of Bcl-xL and Bcl-2 contents was observed in MDA-MB-231 cells.

### 2.9. Effects on Caspases Activity

**Figure 8:**
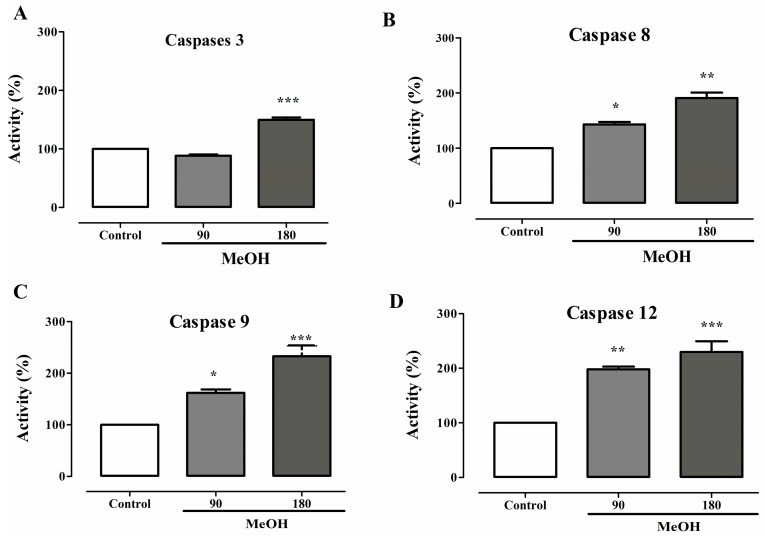
Effects of *F. umbellata* MeOH extract on caspases activities. Activation of caspase-3 (**A**); caspase-8 (**B**); caspase-9 (**C**); and caspase-12 (**D**) by *F. umbellata* MeOH extract. Cells were incubated with 90 and 180 µg/mL for 24 h. Caspases activities were measured by monitoring the cleavage of fluorogenic substrates specific for each caspase. The activity is given as percentage. The results are expressed as the mean ± SEM of three independents experiments. * *p* < 0.05, ** *p* < 0.01 and *** *p* < 0.001 as compared to control.

Caspase-3, caspase-8, caspase-9 and caspase-12 were assessed to determine whether the mitochondrial pathway cascade or the external stimuli is involved in the induction of apoptosis by *F. umbellata* MeOH extract on MDA-MB-231 cells. A significant increase in caspase-3 (50%), caspase-8 (88%), caspase-9 (133%) and caspase-12 (130%) activities was observed following 12 h of *F. umbellata* MeOH extract incubation at the concentration of 180 µg/mL, while only caspases-8 (42%), caspase-9 (62%) and caspase-12 (97%) activities were increased with this extract at the concentration of 90 µg/mL (Figure 8). These results suggest that *F. umbellata* MeOH extract induced apoptosis in MDA-MB-231 cells by increasing caspase-8 and caspase-9 activities.

### 2.10. Effects on Body Weight and Survival

**Figure 9:**
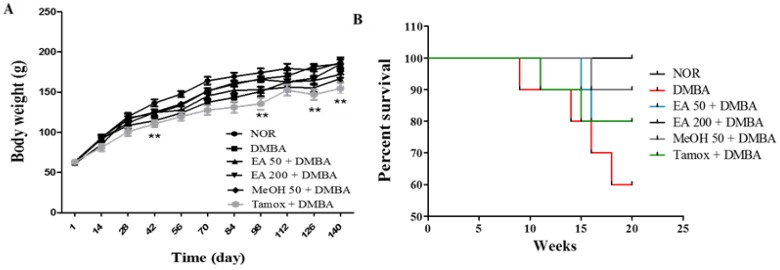
Effects on animal body weight and survival. Body weight (**A**) and Kaplan Meir survival curve (**B**) after 20 weeks of treatment. NOR, Normal control treated with 2% ethanol; DMBA, DMBA control treated with 2% ethanol; EA + DMBA, Animals treated with the *F. umbellata* aqueous extract at the doses of 50 mg/kg and 200 mg/kg. MeOH + DMBA, Animals treated with the *F. umbellata* methanol extract at the dose of 50 mg/kg. TAM + DMBA, Animals treated with tamoxifen (3.3 mg/kg); All groups except of normal group (NOR) received an intragastric dose of DMBA at the dose of 50 mg/kg followed by a 10-day subcutaneous administration of 17β-estradiol (5 mg/kg). Data are represented as mean ± SEM (*n* = 10). ** *p* < 0.01 as compared to negative control.

Figure 9 shows that a 20-week treatment with different substances following DMBA administration did not alter the animal body weights. However, animals of Tamox group presented a significant lower body weight throughout the experiment as compared to normal group (Figure 9A). The Kaplan Meir survival rate curve (Figure 9B) showed that the higher amount of animal’s death throughout the experiment (20 weeks) was recorded in DMBA group (40%), followed by animals treated with tamoxifen (20%) and *F. umbellata* AE extract at the dose of 50 mg/kg (20%).

### 2.11. Effects on Ovarian Tumors

**Figure 10:**
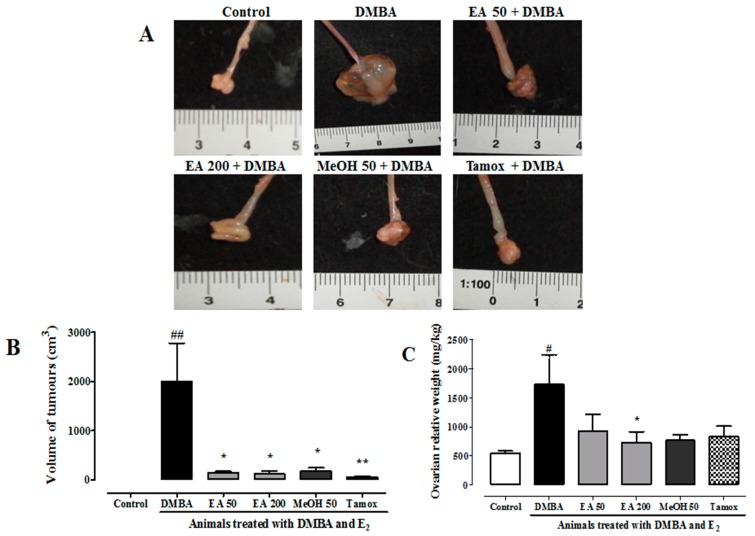
Effects of *F. umbellata* extracts on tumor parameters: ovarian tumors (**A**); volume of tumors (**B**); and ovarian relative weight (**C**) after 20 weeks of treatment. Control, Normal control treated with 2% ethanol; DMBA, DMBA control treated with 2% ethanol; EA + DMBA, Animals treated with the *F. umbellata* aqueous extract at the doses of 50 and 200 mg/kg. MeOH + DMBA, Animals treated with the *F. umbellata* methanol extract at the dose of 50 mg/kg. TAM + DMBA, Animals treated with tamoxifen (3.3 mg/kg). All groups excepting the normal control group (NOR) received an intragastric dose of DMBA at the dose of 50 mg/kg followed by a 10-day subcutaneous administration of 17β-estradiol (5 mg/kg). Data are represented as mean ± SEM (*n* = 10). ^#^ *p* < 0.05 and ^##^ *p* < 0.01 as compared to normal control. * *p* < 0.05 and ** *p* < 0.01 as compared to DMBA control.

In this study, no palpable breast tumor was observed during the 20 weeks of study. However, all animals that received DMBA (50 mg/kg) followed by estradiol treatment (5 mg/kg s.c/10 days) presented at least one ovarian tumor as compared to their age-matched normal group, which did not present any tumors (Table 4 and Figure 10). Negative control animals (DMBA) presented 60% of tumor incidence with a relative tumor burden of 17.32 g. *F. umbellata* AE (200 mg/kg) and MeOH (50 mg/kg) extracts protected animals against ovarian tumors. Significant (*p* < 0.001) inhibition of tumor burden of 58% (*F. umbellata* EA 200 mg/kg) and 46% (*F. umbellata* MeOH 50 mg/kg) was observed. The tamoxifen was used in this study as positive control against breast tumors; however, animals belonging to this group also presented high ovarian tumor incidence (40%) as compared to DMBA groups. Moreover, it was observed a significant inhibition of tumor burden (52%) in this group (Table 4).

Figure 10 depicts the representative pictures of observed ovary tumors in different groups (Figure 10A). The tumor volume and tumor weight measurements showed that *F. umbellata* AE and MeOH extract at all tested doses as well as tamoxifen significantly (*p* < 0.01) prevented tumor growth. This was materialized by a reduction of tumor volume (Figure 10B) and tumor weight (Figure 10C), but a statistical significance was obtained only with *F. umbellata* EA extract at 200 mg/kg in the tumor weight parameter.

### 2.12. Histomorphological Analysis of Estrogen Target Organs

**Figure 11:**
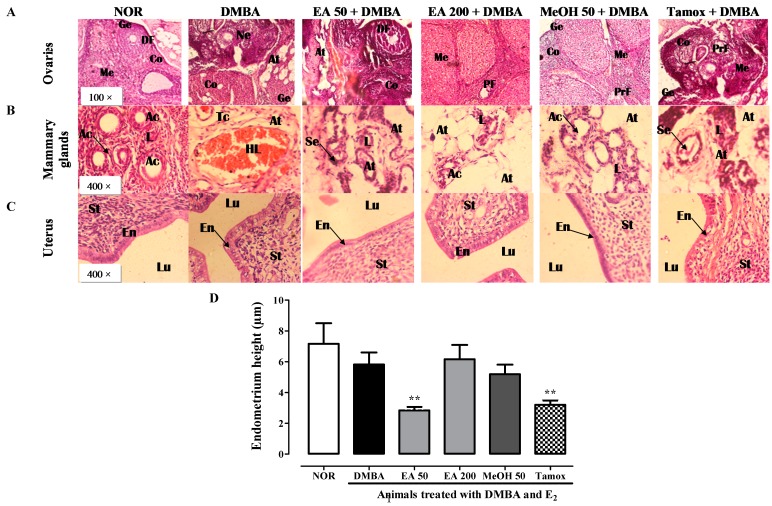
Effects of *F. umbellata* extracts on different organs. Microphotographs H&E (400×) of ovaries (**A**); mammary glands (**B**); and uterine (**C**); as well as graphic representation of endometrium height (**D**) after 20 weeks of treatment. Control, Normal control treated with 2% ethanol; DMBA, DMBA control treated with 2% ethanol; EA + DMBA, Animals treated with the *F. umbellata* aqueous extract at the doses of 50 and 200 mg/kg. MeOH + DMBA, Animals treated with the *F. umbellata* methanol extract at the dose of 50 mg/kg. TAM + DMBA, Animals treated with tamoxifen (3.3 mg/kg). All groups excepting the normal group (NOR) received an intragastric dose of DMBA at the dose of 50 mg/kg followed by a 10-day subcutaneous administration of 17β-estradiol (5 mg/kg). Data are represented as mean ± SEM (*n* = 10). ** *p* < 0.01 as compared to DMBA control. La, lumen of alveoli; At, adipose tissue; Se, eosinophil secretion; L, lobular; HLU, hyperplastic lobular unit; St, stroma, En, endometrium; Lu, lumen of uterine; Ge, germinal epithelium, PF, primordial follicles, PrF, primary follicle, Ne, necrotic and inflammatory cells, DF, Deegraff follicle.

The histomorphological analysis of ovary sections showed a pronounced hyperplasia of ovaries with atypical malignant structure in animal of DMBA group as compared to normal animals (Figure 11A). Animals treated with *F. umbellata* AE (50 mg/kg) extract and tamoxifen presented a structure near to that observed in DMBA group. Animals treated with *F. umbellata* EA (200 mg/kg) and MeOH (50 mg/kg) presented microarchitecture of ovaries protected against hyperplasia.

Although palpable mammary tumors were not observed, it was found many small nodules in mammary fat pad in animals treated with DMBA. Histological sections of mammary tissue from rats exposed to DMBA and estradiol for seven days showed hyperplasia and elongation of duct structure as compared to animals of normal group, which showed normal acini buds (Figure 11B). Atypical structures of mammary gland were also found in animals treated with EA 50. However, *F. umbellata* EA (200 mg/kg) and methanolic (50 mg/kg) extracts as well as tamoxifen have protected animals against DMBA-induced mammary gland hyperplasia.

The histomorphological analysis in uterus (Figure 11C) did not shown endometrial tumors, however, it was observed an increase of mitotic cells in endometrium of animal that received DMBA, exception made for those treated with *F. umbellata* EA (50 mg/kg) and MeOH (50 mg/kg) extracts. The endometrium measurement showed that animals treated with *F. umbellata* EA extract (50 mg/kg) and tamoxifen have a significant (*p* < 0.01) reduction of endometrium (Figure 11D).

### 2.13. Effects of F. umbellata Treatment on Relative Organ Weights

Table 5 depicts the relative organ weights after 20 weeks of treatment with different substances. In line with the above observed reduction of uterine epithelial height, it was noted a decrease of uterine wet weight following tamoxifen (*p* < 0.05) and *F. umbellata* AE extract (50 mg/kg) treatment. Moreover, a significant reduction of relative liver weight in MeOH 50 plus DMBA (*p* < 0.01) and tamoxifen plus DMBA (*p* < 0.05) groups was observed as compared to normal and DMBA groups. *F. umbellata* EA extract at the dose of 200 mg/kg induced a significant (*p* < 0.05) increase of spleen (*p* < 0.01) and adrenal glands (*p* < 0.01) weights as compared to normal and DMBA groups. A significant (*p* < 0.001) increase of brain weight was also observed with animals of tamoxifen group.

### 2.14. Effects of F. umbellata Extracts on Various Toxicological Parameters

**Figure 12:**
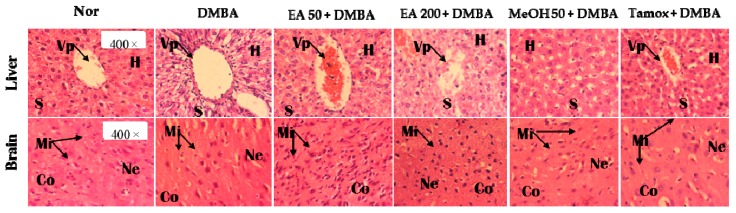
Effects of *F. umbellata* extracts on microphotographs H&E (400×) of liver (**top**); and brain (**bottom**) after 20 weeks of treatment. Control, Normal control treated with 2% ethanol; DMBA, DMBA control treated with 2% ethanol; EA + DMBA, Animals treated with the *F. umbellata* aqueous extract at the doses of 50 and 200 mg/kg. MeOH + DMBA, Animals treated with the *F. umbellata* methanol extract at the dose of 50 mg/kg. TAM + DMBA, Animals treated with tamoxifen (3.3 mg/kg); All groups excepting the normal group (Nor) received an intragastric dose of DMBA at the dose of 50 mg/kg followed by a 10-day subcutaneous administration of 17β-estradiol (5 mg/kg). Data are represented as mean ± SEM (*n* = 10). Vp, portal vein; H, Hepatocyte; S, sinusoids; Mi, microglia; Ne, Neuron; Co, Cortex.

The histomorphological sections of liver (Figure 12A) and brain (Figure 12B) from animals of normal group showed normal microarchitecture. No significant changes in liver and brain histopathological characteristics were noted among various groups. Regarding hematological profile, a slight increase of red blood cell count was observed in animals treated with EA extract at the dose of 50 mg/kg as compared to normal animals (Table 6). Animals treated with tamoxifen presented a significant decrease of mean corpuscular hemoglobin (MCH) (*p* < 0.05) and mean corpuscular hemoglobin concentration (MCHC) (*p* < 0.01) as compared to normal and DMBA group.

### 2.15. In Vivo Antioxidant Activities of F. umbellata Extracts

**Figure 13:**
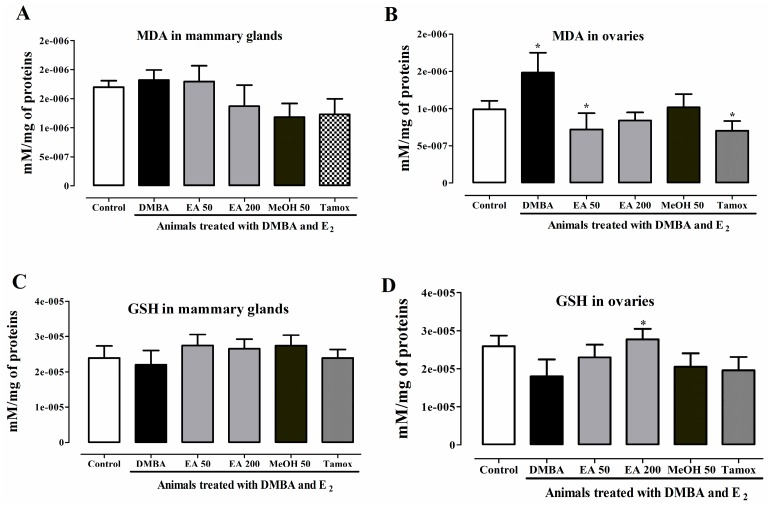
Effects of *F. umbellata* extracts on MDA and GSH levels in different organs. MDA level in mammary glands (**A**) and ovaries (**B**) and GSH level in mammary glands (**C**) and ovaries (**D**) after 20 weeks of treatment. Control, Normal control treated with 2% ethanol; DMBA, DMBA control treated with 2% ethanol; EA + DMBA, Animals treated with the *F. umbellata* aqueous extract at the doses of 50 and 200 mg/kg. MeOH + DMBA, Animals treated with the *F. umbellata* methanol extract at the dose of 50 mg/kg. TAM + DMBA, Animals treated with tamoxifen (3.3 mg/kg). All groups excepting the normal group (NOR) received an intragastric dose of DMBA at the dose of 50 mg/kg followed by a 10-day subcutaneous administration of 17β-estradiol (5 mg/kg). Data are represented as mean ± SEM (*n* = 10). * *p* < 0.05 as compared to negative control.

DMBA also induced carcinogenesis in mammals by oxidative damage. Thus, oxidative stress status in mammary glands and ovarian homogenates was evaluated. An increase in MDA levels was observed in both mammary glands and ovaries of DMBA group as compared to normal group (Figure 13A,B). However, statistical significance was reached only for ovaries. Conversely, a non-significant decrease of GSH level was observed in mammary glands (Figure 13C) and ovaries (Figure 13D). *F. umbellata* AE and MeOH extracts decreased MDA levels in both mammary glands and ovaries but statistical significance (*p* < 0.05) was reached only in EA 50 group such as in tamoxifen group. All treatments induced a non-significant increase in GSH level in mammary glands, while in ovaries only *F. umbellata* AE extract at the dose of 200 mg/kg induced a significant increase in GSH level as compared to DMBA group.

## 3. Discussion

Cytotoxicity assays are useful to indicate the ability of a substance to cause cell death by alteration of one or more cellular functions [20]. Among the available cytotoxicity assays for the detection of cell viability, the Alamar blue (rezasurin) assay is a reliable and reversible assay that measures the ability of mitochondrial function of viable cells to reduce rezasurin in the red-fluorescent resorufin [21]. In this study, the MeOH extract induced greater cytotoxicity than AE extract in all tested cell lines, indicating that some compounds of this extract which is not present in the AE extract could have antitumor activity. The fact that MeOH extract was found to contain more flavonoids, flavonols and alkaloids than AE extract could account for this differential activity, since isoflavons were already described to induce apoptosis [22]. Although the AE extract did not induce significant cytotoxicity, it was observed that both AE and MeOH extracts were less active in estrogen receptor-positive cell (MCF-7) than in estrogen receptor-negative cells (MDA-MB-231 and HCC 1954). *F. umbellata* AE and MeOH extracts have been previously reported to possess estrogenic properties and therefore recognized as potential sources of weak phytoestrogens [19]. Phytoestrogens have been reported to exert two opposite actions in cancer cells depending on their concentrations [23]. At lower concentrations (<10 µM), phytoestrogens, such as genistein, acted as ligand of ERs and stimulated metabolic pathways, which in turn induced ER-positive MCF-7 cells proliferation, but not the ER-negative MDA-MB-231 breast cancer cells. However, at higher concentrations, mechanisms that are not dependent on ER pathway and antioxidant properties of the flavonoids seem to be triggered [23]. The aforementioned arguments might explain why MDA-MB-231 cells seem to be more sensitive than MCF-7 to *F. umbellata* constituents. In contrast, *F. umbellata* MeOH extract, and its soluble dichloromethane fraction showed cytotoxic effects in all tested cell lines with a CC_50_ around 200 µg/mL, whereas the main phytoconstituent 7-methoxycoumarin did not induce any cytotoxic effect up to 500 µM, suggesting that this compound does not contribute to the *in vitro* antitumor activity of the extract. However, we cannot exclude the possibility that 7-methoxycoumarin might contribute to the in vivo antitumor effects of *F. umbellata* extract, since one of its related metabolite 7-hydroxycoumarin was reported to be cytotoxic in several human tumor cell lines [24]. *F. umbellata* MeOH extract displayed a selective index ≥ 2 against the ER-negative breast cancer cell line, which is of interest for the research of alternative breast cancer treatment [25]. However, the United States National Cancer Institute’s plant screening program strongly recommends to follow-up with a crude extract when its CC_50_ after 72 h of incubation is less than 20 µg/mL [26]. In this study, *F. umbellata* MeOH extract appeared to be a weak cytotoxic agent with a CC_50_ of 180 µg/mL in MDA-MB-231 cells after 24 h of incubation. However, considering this plant is used in African traditional system to manage various ailments, we sought to investigate the underlying mechanisms by which it induced cytotoxic effects.

Apoptosis is a regulated multistep pathway, which is responsible for programmed cell death in normal tissues for monitoring cell number and homeostasis. Morphologically, it involves cell shrinkage, chromatin condensation, and nuclear and cell fragmentation, which result in the formation of membrane-enclosed apoptotic bodies containing organelles. Then, the apoptotic bodies are engulfed by phagocytic cells without inflammation occurrence [27]. Although the exact details of cell death pathways are not yet completely elucidated, it is recognized that apoptosis is triggered either by intrinsic or extrinsic mechanisms [28]. The intrinsic apoptosis involves mitochondrial membrane permeability that can be triggered by stress, cellular damage and results eventually in caspase-9 and caspase-3 activation [29,30]. In fact, the internal membrane of mitochondria contains various proteins that participate in different metabolic pathways. Mitochondrial alterations can induce the liberation of molecules, such as cytochrome c, which in turn induce caspase-3 activation and eventually stimulate the apoptotic cascade. It is known that this sequence of events might result in the overproduction of ROS, and/or the decrease the disturbances in the mitochondrial membrane potential [31]. On the other hand, the extrinsic pathways are initiated by ligands binding to transmembrane receptors. Caspase-8 is the principal protease activated by the extrinsic apoptosis pathway, resulting in caspase-3 activation [28].

In this study, *F. umbellata* MeOH extract induced apoptosis in both MCF-7 and MDA-MB-231 cells. It induced DNA fragmentation and chromatin condensation observed in AO/EB staining, but also by cytometry profile. These results are in accordance with reports, which showed that apoptosis can be triggered by extrinsic factors, such as flavonoids [22,32]. The constituents of *F. umbellata* extract could bind death ligands located in the plasmatic membrane, which in turn could activate caspase-8, then would further activate its downstream effector caspase-3 [29]. Here we showed that the caspase-3, caspase-8, caspase-9 and caspase-12 activities were increased after 12 h of incubation with *F. umbellata* extract. The increase in caspase-8 activity by *F. umbellata* extract suggests that the extrinsic apoptosis pathway was activated. However, it is now well established that the extrinsic pathway can crosstalk to the intrinsic pathway through the caspase-8-mediated cleavage of Bax-like BH3 protein (BID), a member of the Bcl-2 family of proteins [33]. Since caspase-9 activity was increased following treatment with this extract, *F. umbellata* constituents might also induce apoptosis by the activation of mitochondrial pathway. These results suggest that *F. umbellata* constituents are promising agents to kill tumor cells since they seem to activate both intrinsic and extrinsic apoptosis pathways.

Of note, many compounds induced apoptosis in cancer cells by increasing intracellular ROS regeneration, forming pores in mitochondrial membrane leading to the release of various apoptogenic molecules [34], including the cytochrome c and Bcl-2 family proteins in mitochondrial transmembrane. Once in the cytosol, cytochrome c binds to protein APAF-1 forming a complex named apoptosome, which mediates the activation of the caspase-9 [35]. Both the activation of caspase-8 and caspase-9 culminate in the activation of effector caspase-3 [36]. Although the expression of cytochrome c was not assessed in this study, it was observed a decrease in mitochondrial transmembrane potential, an increase in intracellular ROS, and a decrease of anti-apoptotic Bcl-2 and Bcl-xL protein contents induced after incubation with *F. umbellata* extract. Overall, these results strongly suggest that *F. umbellata* constituents also trigger the intrinsic pathways of apoptosis. Cancer cells generally overexpress antiapoptotic factors, such as Bcl-2, in order to reduce the intrinsic apoptosis signaling [36]. The ability of *F. umbellata* MeOH extract to decrease the anti-apoptotic Bcl-xL and Bcl-2 proteins expression might inhibit the capacity of cancer cells to ignore apoptosis signal. This hypothesis needs to be further elucidated.

Furthermore, it was observed that the cytotoxic effect of *F. umbellata* MeOH extract concords with the inhibition of MDA-MB-231 cells migration. In fact, cell migration was significantly inhibited as compared to untreated cells and an increased rate of wound healing could be observed at all time points examined. One of the striking results in this study is that *F. umbellata* MeOH extract inhibited MDA-MB-231 cells migration as well as cells invasion, which can be related to its ability to induced apoptosis in this cell line. Numerous natural substances have been reported to inhibit cell migration by inducing cell cycle arrest or by inhibiting matrix metalloprotease-2 and -9 (MMP2 and MMP9) activities [37]. *F. umbellata* constituents did not induce changes in MDA-MB-231 cells cycle. However, the possible inhibition of MMP2 and MMP9 needs to be further investigated to better understand the observed results. Nevertheless, our results seem to have a valuable significance, since cell migration and invasion are important steps required for breast cancer metastasis [38].

Based on the in vitro antitumor activities of *F. umbellata*, we sought to investigate its in vivo chemopreventive potential, since animal models offer a possibility to compare effect of the substance on normal and neoplastic tissues. The DMBA, an organic environmental pollutant generated from incomplete combustion of fossil fuels, was used for this purpose. It is present in tobacco and various foods and now recognized to cause mammary tumors in humans [39]. This compound is extendedly used to induce cancer in rodent, mainly mammary tumors, because the tumors typically developed in exposed rats closely mimic those of human breast cancer [40]. In this study, a single intragastric dose of 50 mg/kg of DMBA followed by a 10-day administration of 17-β estradiol were used to induce mammary tumors in Wistar rats. No palpable breast tumors were observed after 20 weeks of experiment. However, the histopathological analysis of mammary section revealed ductal hyperplasia in rats exposed to DMBA and *F. umbellata* AE extract (50 mg/kg) treated rats. These results are in line with numerous scientific reports, which showed that DMBA alters the normal process of mammary gland differentiation [40,41,42]. Animal treated with *F. umbellata* AE (200 mg/kg) and MeOH (50 mg/kg) extracts as well as tamoxifen were protected against mammary glands hyperplasia. Since hyperplasia has been reported to be close to subsequent steps leading to neoplasia [40], the observed chemoprotective effect is an encouraging result. Tamoxifen is an effective antiestrogen for the treatment of advanced breast cancer [43], which can explain its protective effect against mammary hyperplasia.

As can be observed in the results 60% of rats of DMBA group developed ovarian tumors. Of note, ovarian tumors were reported to be induced in rodents by intragastric administration of DMBA [44]. Our findings are consistent with various investigators that found ovarian tumors in rodents following exposure to DMBA [45]. Jull et al. [46] reported that a single dose of 5 mg of DMBA given by intragastric administration gave rise to development of ovarian tumors, while at dosage levels not higher than 0.25 mg, the compound failed to induce any tumor formation in animals. Taguchi et al. [47] reported that neonatal treatment with estrogen followed by adult treatment with DMBA induced rapid ovarian tumorigenesis. This study is similar to our findings since, after DMBA administration, animals were exposed to high amount of estradiol (5 mg/kg/10 days) in this study. It was also observed that only 60% of rats belonging to DMBA group developed ovarian tumors. These findings are in accordance with scientific reports, which showed that DMBA treatment does not always successfully produce ovarian carcinoma; the rate of incidence is approximately 50% and the reason for this failure remains unclear [46,47]. Interestingly, animals treated with *F. umbellata* extract were protected against the incidence and burden of ovarian tumors.

*F. umbellata* constituents might act, as observed in vitro, by a mitochondrial dysfunction pathway to induce apoptosis and inhibit tumor growth. Many plant-derived polyphenolic compounds have been identified to have potential cancer preventive or therapeutic effects. This includes a variety of flavonoid molecules and among the flavonoids there are several flavonols that are of interest, such as quercetin. *F. umbellata* was found rich in flavonoids and mainly flavonols, which may be responsible at least in part of its observed in vitro cytotoxic effects and in vivo chemopreventive effects. Indeed, an anticancer role for flavonoids has been intensively reviewed [48]. Data from the Nurses’ Health Study indicated that participants in the highest quintile of flavonol intake had modestly lower risk of ovarian cancer than did participants in the lowest quintile [49]. *F. umbellata* extracts contains potential anticancer active ingredients of different functional and structural properties, such as antioxidants (flavonoids).

Plant extracts consist of a mosaic of compounds displaying more than one mode of action on several targets and therefore might be better treatment option than synthetic drugs. Flavonoids form the largest group of natural phenolic compounds and possess excellent free radical scavenging and antioxidant properties. *F. umbellata* extract is well known as phytoestrogen [19]. It is well established that at the concentration 100- to 1000-fold superior, phytoestrogens can enter in competition with endogen estrogens for ERs [50]. This can account for the antiestrogenic activity observed for *F. umbellata* extract on uterus in this study. Knowing that anti-estrogenic effects are needed effect on estrogen-dependent tumors, we can hypothesize that flavonoids, mainly flavonols detected in *F. umbellata* extract, might influence estrogen-dependent pathway to inhibit tumor cells proliferation. Another mechanism by which *F. umbellata* might induce its antitumor activity may involve antioxidant properties. Indeed, in order to minimize the damage of ROS, aerobics organisms are endowed with enzymatic and non-enzymatic antioxidant defenses. One of the principal enzymatic defenses against hydrogen peroxide (H_2_O_2_) is glutathione peroxidase [51]. GSH is the most important non-enzymatic antioxidant, and normally the majority of the molecules of GSH remain reduced [52]. In contrast, MDA represents a lipid peroxidation marker. The main ROS target is the polyunsaturated fatty acid in cell membranes. They cause lipid peroxidation and formation of MDA, which may lead to damage the cell structure and function [53]. The chemopreventive properties of *F. umbellata* extract might also be related to its antioxidant activity, since it increased GSH content and reduced MDA level as compared to DMBA group. In addition, the antioxidant actions of flavonols are implicated in cancer chemoprevention [41].

## 4. Materials and Methods

### 4.1. Chemicals and Reagents

Serums and antibiotics were purchased from GIBCO (Grand Island, NY, USA). The 17β-estradiol benzoate [(Estr-1,3,5(10)-trien-3,16α,17β-triol), purity ≥ 98%] was obtained from Sigma-Aldrich (Hamburg, Germany). The 2-[4-(2-hydroxyethyl)piperazin-1-yl]ethane sulfonic acid (HEPES, purity ≥ 99.5%) was purchased from Ludwig Biotecnologia Ltd. (Alvorada, RS, Brazil). DMBA (purity ≥ 95%) was purchased from Sigma-Aldrich (Stanford, Germany). Trypan blue (0.4%), Alamar blue, acridine orange, ethidium bromide and cell culture media were purchased from Sigma-Aldrich (St. Louis, MO, USA). Tamoxifen citrate (Mylan^®^) was purchased from MYLAN SAS (Saint-Priest, France). The JC-1 probe (5,5′,6′,6-tetrachloro-1,1′,3,3′-tetraethyl benzymidazol carbocianyne iodide) and DCFH-DA (2′,7′-dichlorofluorescein diacetate) were from Invitrogen (Carlsbad, CA, USA). Millicell^®^ cell culture inserts were purchased from Merck Millipore Ltd. (Tullagreen Carrigtwohill, Ireland). The specific antibodies were purchased from Santa Cruz Biotechnology Inc. (Santa Cruz, CA, USA). The ApopNexin™ FITC Apoptosis Detection Kit was purchased from Millipore (Billerica, MA, USA). Ultrapure Milli-Q water was used to prepare all solutions and buffers in all experiments.

### 4.2. Plant Material

Stem barks of *F. umbellata* were harvested in Yaounde (Cameroon), at the geographical coordinates 32 N0778863, E0428160 ± 23 m (“GARMIN” GPS), in September 2013. The botanical sample used was identified and authenticated by Mr. Victor Nana, Botanist at the National Herbarium of Cameroon (HNC) in Yaounde and a voucher specimen (number 99/HNC) was deposited at the HNC.

### 4.3. Preparation of Extracts and Isolation of 7-Methoxycoumarin

After drying and grinding the stem bark of *F. umbellata*, 2000 g of the powder were macerated in water at room temperature (5 L of solvent × 3, 48 h per extraction). The combined solutions were filtered with Whatman paper No. 4 and evaporated using an oven with ventilation (40 °C, 48 h) to yield 229.8 g of aqueous extract (AE). Further, another 2700 g of the powder were macerated in 95% methanol at room temperature (5 L of solvent × 3, 48 h per extraction). The combined solutions were evaporated under reduced pressure (337 mbar at 40 °C) using a rotary evaporator to yield 162 g of a methanol extract (MeOH). The comparative HPLC chromatograms of *F. umbellata* aqueous and methanol extracts showed that 7-methoxycoumarin is the major compound each extract. The 7-methoxycoumarin was then isolated from *F. umbellata* methanol extract as previously reported [19].

### 4.4. Preliminary Phytochemical Investigations

The phytochemical screening of *F. umbellata* aqueous and methanol extracts was performed according to the method described by Odebiyi and Sofowora [54]. Concentrations of total phenols, flavonoids, flavonols and alkaloids were measured according to methods described by Zhishen et al. [55], Makkar et al. [56], Zhishen et al. [55] and Hazra et al. [57], respectively.

### 4.5. Cell Lines

The MCF-7 (human estrogen receptor (ER)-positive breast adenocarcinoma cells), MDA-MB-231 (human ER-negative breast adenocarcinoma cells), SK-MEL-28 (human melanoma cells), HUVEC (human umbilical vein endothelium cells), MCR-5 (human fetal lung fibroblast cells), 4T1 (mouse mammary tumor cells), HCC-1954 (human ER-negative, PR-negative and HER2-positive breast cancer cells), SF-295 (human glioblastoma cells) and NIH/3T3 (murine fibroblast cells) were obtained from the Rio de Janeiro Cell Bank (Federal University of Rio de Janeiro, Rio de Janeiro, Brazil).

### 4.6. Cell Culture

MDA-MB-231, SK-MEL-28 and MRC-5 cells were cultured in Dulbecco’s Modified Eagle Medium (DMEM) medium supplemented with 10% of fetal bovine serum (FBS). MCF-7, HUVEC, NIH/3T3, SF-295 and 4T1 cells were cultured in RPMI-1640 medium supplemented with 10% FBS HCC-1954 cells were cultured in RPMI-1640 medium supplemented with 10% FBS, 5% pyruvate and 10% glucose. All cell cultures were also supplemented with 100 U/mL penicillin, 100 µg/mL streptomycin and 10 mM HEPES. The cell cultures were maintained at 37 °C in a 5% CO_2_ humidified atmosphere and pH 7.4. Every two days, cells were passaged by removing 90% of the supernatant and replacing it with fresh medium. Before performing all experiments, the number of viable cells was assessed by the trypan blue method using a Neubauer chamber.

### 4.7. Animals

Seventy prepubescent female Wistar rats, aged 35–40 days at the start of experiment and weighing around 45–60 g, were supplied by the breeding facility of the Laboratory of Animal Physiology, University of Yaounde I (Yaounde, Cameroon). These rats were housed in plastic cages in groups of five at room temperature. The animals had free access to drinking water and to a standard pellet rat diet. The composition of animal diet was: corn (40%), soy bean (14%), bone flour (3%), wheat (20%), fish flour (12%), crushed palm kernel (4%), sodium chloride (0.82%), peanuts (6%) and vitamin complex (Olivitazol^®^, 0.25%). Rats were treated in accordance with the guidelines and procedures of animal bioethics of the Cameroon Institutional National Ethic Committee (CEE Council 86/609), which adopted all procedures recommended by the European Union on the protection of animals used for scientific purposes. Efforts were made to minimize animal pain and suffering.

### 4.8. Cell Viability Assay

Cytotoxicity of *F. umbellata* extracts (aqueous and methanolic) as well as 7-methoxycoumarin were initially tested at concentrations of 50 and 300 µg/mL in two tumoral (MCF-7 and MDA-MB-231) and one non-tumoral (NIH/3T3) cell lines using Alamar Blue (resazurin) assay as described by O’Brien et al. [21]. This assay evaluates the mitochondrial production as a measurement of cell viability. Extracts that inhibiting more than 50% growth were therefore investigated for CC_50_ determinations in all studied cell lines. For this, a density of 1 × 10^4^ cells/well in 100 µL of culture medium was seeded in a 96-well plate and allowed to adhere overnight. After 24 h, cells were exposed to different substances at concentrations ranging between 50 to 300 µg/mL for extract and fraction and 50 to 500 µM for 7-methoxycoumarin. The fluorescent intensity was determined by a Perkin Elmer LS55 spectrofluorimeter (Becton Dickinson, San Jose, CA, USA) with excitation at 530 nm and emission at 590 nm. The cytotoxic concentration which kill 50% of cells (CC_50_) was determined by nonlinear regression analysis of the logarithm of concentration in function of the normalized response (percentage of cell viability) using the software GraphPad Prism 6.0 (GPW6-242831-RBMZ-03274). Each experiment was performed in triplicate and repeated three times.

### 4.9. Morphological Identification for Cell Death

The Acridine Orange (AO, 3,6-dimethylaminoacridine), a nucleic acid selective metachromatic and ethidium bromide (EB, 3,8-diamino-5-ethyl-6-phenylphenanthridinium bromide) stains were used to qualitatively characterized cell death mechanism. For this, MCF-7 and MDA-MB-231 cells (3.5 × 10^5^ cells/mL) were plated in a 12-well plate and treated with *F. umbellata* MeOH extract at 45 (CC_13_), 90 (CC_25_) and 180 µg/mL (CC_50_) or solvent control (DMSO) for 24 h. After incubation, medium was removed and cells were washed with phosphate-buffered saline (PBS) and dye mixture (0.3 µg/mL AO and 1 µg/mL EB) was added in each well. Cells were immediately viewed using a Nikon eclipse TS100 inverted microscope at 400× magnifications with excitation filter 480/30 nm; dichromatic mirror cut-on 505 nm LP; and barrier filter 535/40 nm. Pictures were taken with a Nikon COLPIX digital camera and analyzed by image editor software (ImageJ^®^).

### 4.10. Cell Death Mechanism by Cytometry

For the evaluation of cell death (necrosis/apoptosis) mechanism, cells were labeled with Annexin FITC-conjugated (1:500) and propidium iodide (PI) fluorochrome. Briefly, MCF-7 and MDA-MB-231 cells (3.5 × 10^5^ cells/mL) were plated in a 12-well plate and treated with *F. umbellata* MeOH extract at 45, 90 and 180 µg/mL or solvent control (DMSO) for 24 h. Further, cells were washed twice with cold PBS (500 mL/well) and resuspended in the binding buffer. The pellets were stained with the fluorescent probe solution (50 mg/mL PI and 1 mg/mL Annexin in PBS) on ice in dark for 15 min. Cells were analyzed by flow cytometer BD DACS Verse (Becton Dickinson, Franklin Lakes, NJ, USA) and the results were analyzed using WinMDI 2.9 software. Each experiment was repeated three times.

### 4.11. Cell Cycle Analysis

MCF-7 and MDA-MB-231 cells (3.5 × 10^5^ cells/mL) were plated in a 12-well plate for 24 h. Then, the medium was replaced and *F. umbellata* MeOH extract at 45, 90 and 180 µg/mL or solvent control (DMSO) were added for 24 h incubation. Cells were washed many times with cold PBS and resuspended in 70% ice ethanol and stored at −20 °C for fixation. After 30 min, the mixture of PBS with 2% bovine serum albumin (BSA) was added. The cell pellets obtained after centrifugation, were washed and permeabilized with lysis buffer (0.1% Triton X-100 and 100 µg/mL RNAse) and staining with 20 µg/mL PI). Cell deoxyribonucleic acid (DNA) content in the different cell cycle phases was determined by flow cytometry (BD DACS Verse, Becton Dickinson, Franklin Lakes, NJ, USA), and the results were analyzed using WinMDI 2.9 software. Each experiment was repeated three times.

### 4.12. Wound-Healing Assay

This assay was performed to assess the ability of *F. umbellata* constituents to inhibit MDA-MB-231 cells migration using the scratch method. Briefly, cells were plated in the 12-well plates at a density of 3 × 10^5^ cells/mL in DMEM with 10% FBS. Twenty-four hours after, the cell layer was scratched with a sterile plastic tip and then washed twice with PBS to remove cells mechanically detached. The cells were then maintained in DMEM for 48 h with *F. umbellata* MeOH extract at 45, 90 and 180 µg/mL or solvent control (DMSO). The cell migration was recorded using a light microscopy Nikon Eclise TS100 (Melville, NY, USA), photographed every 12 h and area of wound healing was evaluated by image editor software (ImageJ^®^). Each experiment was repeated three times.

### 4.13. Cell Invasion Assay

For this assay, Matrigel pre-coated Millicell culture inserts (8 pores 8.0 μm, diameter 10 mm) were used. Inserts were washed twice with DMEM and after rehydrated for 30 min in DMEM before the experiment. Further, 50 µL of matrigel (1:10 in DMEM without serum) was added in the inserts contained in 24 well plates. The insert with matrigel was maintained at 37 °C for 1 h in order to polymerize. A suspension of 5 × 10^4^ MDA-MB-231 cells in 200 μL was homogenously added in the upper chamber and the lower chamber was filled with DMEM containing 10% FBS. Cells were allowed to invade for 48 h at 37 °C, 5% CO_2_ in the absence (DMSO) or in presence of *F. umbellata* MeOH extract at 90 and 180 µg/mL. Cells that did not migrate or invade were removed using a cotton bud whereas cells that had migrated or invaded were fixed with glutaraldehyde 5% and stained with crystal violet solution 0.1% for 10 min. Pictures were taken with a Nikon COLPIX digital camera and a minimum of five fields was counted per insert by image editor software (ImageJ^®^). Each experiment was performed twice and the average of cells/fields was calculated.

### 4.14. Measurement of the Mitochondrial Transmembrane Potential

The mitochondrial transmembrane potential was evaluated using the lipophilic cationic fluorochrome 5,5′,6′6-tetrachloro-,1′,3,3′tetraethylbenzymidazolcarbocianyne iodide (JC-1). Briefly, MDA-MB-231 cells (3.5 × 10^5^ cells/mL) were seeded in 12-well plates and incubated for 24 h. After 6 h of treatment with *F. umbellata* MeOH extract at 90 and 180 µg/mL or solvent control (DMSO) or positive control (uncoupler CCCP), the electron transport chain JC-1 (10 μg/mL) was added and cells were incubated for 30 min at 37 °C (5% CO_2_). Further, cells were washed twice with PBS and resuspended in PBS. Fluorescence was measured using a Perkin Elmer LS55 spectrofluorimeter (Becton Dickinson, San Jose, CA, USA). JC-1 was excited at 488 nm, the red emission fluorescence was detected at 590 nm and the green fluorescence was detected at 525 nm. Each experiment was performed twice and the mitochondrial potential was presented as a ratio of 590/525 fluorescence and compared with the control cells that were considered to have 100% mitochondrial membrane potential.

### 4.15. Reactive Oxygen Species (ROS) Detection

Intracellular free radical formation was determined using 2′,7′-dichlorodihydrofluorescein diacetate (DCHF-DA) which is oxidized to dichlorofluorescein (DCF) in presence of ROS. For this, MDA-MB-231 cells were seeded into 12-well plates at a density of 3.5 × 10^5^ cells/mL and incubated for 24 h. The medium was removed and replaced with *F. umbellata* MeOH extract at 90 and 180 µg/mL or solvent control (DMSO) for 12 h. Further, cells were covered and incubated with of medium containing fluorescent dye 10 μM DCHF-DA. The treated cells were then washed four times with cold PBS, collected by trypsinization and centrifugation at 600× *g* for 10 min. The cell pellets were then suspended in PBS-EDTA and the DCF fluorescence signal was measured using a spectrofluorimeter Perkin-Elmer LS55 (Becton Dickinson, San Jose, CA, USA) with excitation at 485 nm and emission at 535 nm. The results obtained as fluorescence units were expressed as percentage of ROS, compared with non-treated cells and normalized by the total protein quantity measured by the adapted method of Lowry [58].

### 4.16. Determination of Caspase Activities

To determine the activity of caspase-3, caspase-8, caspase-9 and caspase-12, 2 × 10^6^ cells were incubated with *F. umbellata* MeOH extract at concentrations of 90 and 180 µg/mL for 8 h at 37 °C. Cells were washed twice with PBS and lysed in a buffer containing 50 mM HEPES, pH 7.4, 1 mM phenylmethylsulfonylfluoride (PMSF), 1 µg/mL pepstatin A, 1 µg/mL leupepin, 5 µg/mL aprotinin, 5 mM 3-[(3-cholamidopropyl)-dimethylammonio]-1-propanesulfonate (CHAPS) and 5 mM dithiothreitol (DTT) at 4–8 °C for 20 min. Then, the extract was added to a buffer reaction containing 20 mM HEPES, pH 7.4, 0.1% CHAPS, 2 mM EDTA, 5% sucrose and 5 mM DTT. The reaction medium was supplemented with fluorogenic substrates for each caspase individually as follow: 100 µM Ac-LEHD-AFC for caspase-9, 50 µM Ac-DEVD-AMC for caspase-3, 25 µM Ac-IETD-AMC for caspase-8 or 25 µM Ac-ATAD-AFC for caspase-12. After incubation at 37 °C for 2 h, the caspases activities were monitored using spectrofluorimeter (Perkin Elmer LS55) by the production of fluorescent 7-amino-4-methyl coumarin (AMC) or 7-amino-4-trifluoromethylcoumarin (AFC). Protein content was determined as mentioned earlier. Caspase activity was presented as a percentage taking into account the values of fluorescent units per µg of protein.

### 4.17. Western Blot Analysis

MDA-MB-231 cells (1 × 10^7^ cells/well) were seeded on a 6 well plate with 2 mL of medium containing FBS 10%. After 24 h, *F. umbellata* MeOH extract were added at concentration of 90 and 180 µg/mL and incubated for 24 h. The MDA-MB-231 cells were then harvested in lysis buffer, sonicated, centrifuged for 10 min at 10000× *g* in order to extract total proteins. The total protein concentration of the supernatant was determined as already mentioned earlier. Sodium dodecyl sulfate-polyacrylamide gel electrophoresis (SDS-PAGE) with a 15% gel was carried out loading aliquots containing 50 µg of total protein from each sample and transferred to nitrocellulose membranes. The membranes were blocked with bovine serum albumine (BSA) 5% in Tris-buffered saline with Tween 20 °C (TBS-T) and incubated overnight at 4 °C with primary human specific Bcl-2, Bcl-xL or β-actin monoclonal antibodies at a 1:1000 dilution in TBS-T containing BSA 2.5%. Further, the membranes were washed four times with TBS-T and incubated with anti-mouse IgG peroxidase secondary antibody (1:10,000) (Sigma Aldrich, St. Louis, MO, USA). Immune complexes were visualized by chemiluminescent detection using Amersham ECL™ (GE Healthcare, UK) detected by ChemiDoc MP (Bio-Rad, CA, USA). Proteins were quantified on Bio-Rad image analysis software (Bio-Rad, Hercules, CA, USA) and β-actin was used to ensure equal loading of proteins and normalize the proteins content.

### 4.18. DMBA- Induced Carcinogenesis in Rats

After 10 days of acclimatization, rats aged 45–50 days were randomly assigned to 6 groups of 10 animals in each. Group I served as normal control and Group II as DMBA control. Both groups received the vehicle (ethanol/distilled water: 2/98). Group III served as tamoxifen control and received the ER modulator tamoxifen (Mylan^®^) at the dose of 3.3 mg/kg. Group IV and Group V were treated with *F. umbellata* AE extract at doses of 50 and 200 mg/kg, respectively. The remaining group (group VI) was treated with *F. umbellata* MeOH extract at a dose of 50 mg/kg. All treatments were performed by intra-gastric gavage one week before DMBA administration, once by day around 2:00 p.m., and lasted for 5 months. Carcinogenesis (ovarian tumors) was induced in Group II to Group VII by a single oral dose of DMBA (50 mg/kg) dissolved in 1 mL of olive oil, while Group I received olive oil only. After 5 days, the DMBA action was promoted by the administration of 17β-estradiol benzoate (5 mg/kg, s.c.) for 10 days. Animals were weighed weekly and palpated twice a week to check the development of palpable tumors from the first day of acclimatization until the end of the experiment. Ovarian tumors were detected as a nodule under peritoneal muscle and the time of tumor appearance was recorded. Animals that died during the experiment were autopsied, and those becoming moribund were euthanized for examination. At the end of the 140 days of treatment, all survivors were euthanized under ketamin and valium anesthesia (10 and 50 mg/kg, respectively, i.p.) after a 12 h overnight fasting. One part of the blood sample was collected in EDTA-coated tubes for haematological analysis and the other in dry tubes and centrifuged at 600× *g* for 15 min. After blood collection, the skin was dissected out to expose tumors and all tumors were removed, counted and weighed. The size of these tumors was measured using a 1 mm precision sliding caliper (IGAGING^®^). The tumor incidence rate of each group has been recorded and the tumor volumes were calculated using the following formula: length × weight × height × π/6 [59]. Estrogen target organs (ovaries, uterus, and mammary glands), as well as femur, brain, liver, lungs, spleen, kidneys and adrenergic glands were removed and weighed. A piece of the left ovary and mammary gland tissues of each animal was weighted, homogenized in 0.1 mM phosphate buffer solution and centrifuged at 600× *g* for 15 min. The serum and homogenates were kept at −15 °C for hematological and biochemical parameter analysis. All organs were fixed in 10% neutral formalin solution for histological analysis.

### 4.19. Histological Analysis

Histomorphological changes in mammary glands, ovaries, uterus, brain and liver were determined by using 5 μm tissue sections of paraffin-embedded organs stained with hematoxylin and eosin. Histological sections were observed and images were captured using the complete Zeiss equipment consisting of a microscope Axioskop 40 connected to a computer where the images were transferred and analyzed with the MRGrab 1.0 and Axio Vision 3.1 softwares, all provided by Zeiss (Hallbermoos, Germany). The mammary tumors were classified using the well characterized criteria [40].

### 4.20. Biochemical Analysis

For biochemical analysis, the determination of total protein levels was performed in reference to colorimetric methods described by Gonal et al. [60]. Superoxide dismutase (SOD) activity, malondialdehyde (MDA) level were also measured following the methods of Misra [61] and Wilbur et al. [62], respectively.

For hematological analysis, white blood cell count, lymphocytes, monocytes, granulocytes, red blood cells (RBC) count, hematocrit (Ht), hemoglobin (Hb), mean corpuscular volume (MCV), mean corpuscular hemoglobin (MCH), mean corpuscular hemoglobin concentration (MCHC) and platelet count were evaluated using a Humacount 30^TS^ Automated Hematology Analyzer from Human Diagnostics Worldwide (Wiesbaden, Germany).

### 4.21. Statistical Analysis

For in vitro experiments, the results were presented as mean ± standard error of mean (SEM) of triplicates from three-independent experiments. For in vivo experiment, the data from each experimental group was expressed as mean ± SEM. One-way analysis of variance (ANOVA) followed by Dunnett’s post-hoc test for multiple comparisons were used for statistical analysis of data using GraphPad Prism software version 6.00. Differences were considered significant at a probability level of 5% (*p* < 0.05).

## 5. Conclusions

In summary, this study provides experimental evidence for the first time that *F. umbellata* methanol extract exhibits cytotoxic activity toward a panel of cancer cell lines. It induced apoptosis in both MCF-7 and MDA-MB-231 cells. In MDA-MB-231 cells, the induction of apoptosis was mediated through the activation of caspase-3, caspase-8, caspase-9, caspase-12, and ROS-mediated mitochondrial dysfunction pathway, which was accompanied by the overexpression of Bcl-2 and Bcl-xL proteins. In vivo, *F. umbellata* AE (200 mg/kg) and MeOH (50 mg/kg) extracts significantly reduced ovarian tumor incidence (10%), total tumor burden (58% and 46%, respectively), and average tumor weight (57.8% and 45.6%, respectively) as compared to DMBA control group. These extracts also showed a moderate hyperplasia of mammary glands as compared to DMBA group, suggesting that this plant can help people to fight against cancer. Further investigations are needed to understand the precise mechanism by which *F. umbellata* induced cytotoxic effect. The next step is to perform in-depth phytochemical studies to isolate and characterize active principles of *F. umbellata*.

## Figures and Tables

**Table 1:** General information on 7-methoxycoumarin isolated from *F. umbellata*.

Chemical Names	Crystal Color	Structure, Molecular Weight and Formula
7-Methoxycoumarin Methylumbelliferone 7-Methoxy-2H-chromen-2-one	White	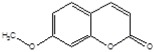 Molecular weight = 176.1 M Molecular formula = C_10_H_8_O_3_

**Table 2:** Quantitative analyses of selected phytochemicals present in *F. umbellata* extracts.

N°	Phytochemical Class	Concentration of *F. umbellata* Extract	
		Aqueous	Methanolic
1	Total phenols	540.21 ± 4.42	651.31 ± 12.03
2	Flavonoids	268.25 ± 35.55	545.33 ± 99.39
3	Flavonols	78.22 ± 6.11	180.15 ± 10.74
4	Alkaloids	60.21 ± 14.38	120.21 ± 6.04

Total phenols, flavonoids and flavonols are expressed in mg eq quercetin/g of dried extract while alkaloids content is expressed in mg eq berberin/g of dried extract. Data are represented as mean ± standard error of mean (SEM) of triplicates from at least three independent experiments.

**Table 3:** CC_50_ values of *F. umbellata* extracts in tumoral and non-tumoral cell lines.

**CC_50_ (µg/mL or µM)**							
**A**		MeOH	AE	FU-Hex	FU-DCM	FU-R	MC
	MCF-7	250	>300	>300	197	>300	>300
	MDA-MB-231	180	>300	190	180	>300	>300
	NIH-3T3	297	>300	270	215	>300	>300
**CC_50_ (µg/mL)**							
**B**		SF-295	4T1	HUVEC	MRC-5	SK-MEL-28	HCC 1954
	MeOH	237	283	449	378	185	192
	FU-DCM	252	245	440	370	176	162
**Selectivity Index**							
**C**		HUVEC/MCF-7		MRC-5/MDA-MB-231		NIH-3T3/4T1	
	MeOH	1.6		2.08		1.05	
	FU-DCM	2.23		2.05		0.88	

CC_50_, Concentration of *F. umbellata* extracts which results in 50% of cell viability; Selectivity Index is equal to CC_50_ of *F. umbellata* extracts on non-tumoral cell lines (NIH/3T3, MCR-5 and HUVEC) divided by CC_50_ determined for cancer cells (MCF-7, MDA-MB-231 and 4T1). (**A**) MCF-7, MDA-MB-231 and NIH-3T3 cells were exposed to *F. umbellata* methanol (MeOH) and aqueous extracts (AE); hexane (FU-Hex), dichloromethane (FU-DCM) and residue (FU-R) fractions from *F. umbellata* MeOH extract as well as 7-methoxycoumarin (MC) at two concentrations (100 and 300 µg/mL or 100 and 300 µM) for the screening; (**B**) CC_50_ of MeOH extract and FU-DCM fraction in SF-295, 4T1, HUVEC, MCR-5, SK-MEL-28 and HCC 1954 cell lines; **C**, selectivity index of MeOH and FU-DCM in human and murine cell lines.

**Table 4:** Ovarian cancer chemopreventive activity of *F. umbellata* extracts after 20 weeks of treatment.

Items	Control	DMBA	EA 50 + DMBA	EA 200 + DMBA	MeOH 50 + DMBA	Tamox + DMBA
Number of rats with tumors/total rats	0/10	6/10	4/10	1/10	1/10	4/10
Tumor incidence (%)	0	60 ^###^	40 *	10 ***	10 ***	40 *
Average tumor weight (g)	-	1.73 ± 0.51	1.60 ± 0.95	0.73 ± 0.18	0.94 ± 0.27	0.83 ± 0.19
% Inhibition related to tumor weight	-	-	7.5	57.8	45.6	52
Total tumor burden (g)	0	17.32	16.04	7.30	9.39	8.28
% Inhibition related to tumor burden	-	-	7	58	46	52

Control, Normal control treated with 2% ethanol; DMBA, DMBA control treated with 2% ethanol; EA + DMBA, Animals treated with the *F. umbellata* aqueous extract at the doses of 50 and 200 mg/kg. MeOH + DMBA, Animals treated with the *F. umbellata* methanol extract at the dose of 50 mg/kg. TAM + DMBA, Animals treated with tamoxifen (3.3 mg/kg); All groups excepting the normal group (NOR) received an intragastric dose of DMBA at the dose of 50 mg/kg followed by a 10-day subcutaneous administration of 17β-estradiol (5 mg/kg). Data are represented as mean ± SEM (*n* = 10). * *p* < 0.05 and *** *p* < 0.001 as compared to DMBA control; ^###^ *p* < 0.001 as compared to normal control.

**Table 5:** Effects of *F. umbellata* extracts on relative weight of various organs after 20 weeks of treatment.

Organs (mg/kg)	Control	DMBA	EA 50 + DMBA	EA 200 + DMBA	MeOH 50 + DMBA	Tamox + DMBA
Uterus	2703.87 ± 494.85	1887.56 ± 212.11	1618.97 ± 73.79	2158.32 ± 305.66	2566.01 ± 418.52	643.38 ± 25.45 *
Liver	33,978.23 ± 608.21	36,983.76 ± 496.00	35,057.02 ± 1189.18	33,073.22 ± 1330.93	31,154.28 ± 535.92 **	32,068.64 ± 1801.93 *
Lungs	7152.24 ± 548.77	6374.34 ± 263.17	6710.31 ± 540.57	7295.43 ± 488.55	6547.02 ± 426.91	6332.43 ± 347.95
Spleen	2115.80 ± 113.88	2021.21 ± 100.23	1964.29 ± 70.05	2841.28 ± 204.94 **	1903.22 ± 50.42	2389.72 ± 139.20
Adrenals	256.92 ± 21.38	260.86 ± 19.99	297.01 ± 30.53	379.80 ± 36.63 *	348.27 ± 36.42	344.08 ± 20.18
Kidneys	4993.25 ± 103.85	5500.42 ± 82.00	4952.97 ± 501.31	5709.14 ± 114.13	5208.32 ± 113.03	5753.71± 236.31
Femur	2734.37 ± 95.99	2990.48 ± 106.61	3019.59 ± 120.11	3013.05 ± 132.31	3262.95 ± 114.77	3163.50 ± 283.44
Brain	8426.09 ± 189.21	8580.18 ± 171.83	9045.49 ± 247.90	8900.86 ± 210.99	8309.45 ± 147.89	10,456.47 ± 376.26 ***

Control, Normal control treated with 2% ethanol; DMBA, DMBA control treated with 2% ethanol; EA + DMBA, Animals treated with the *F. umbellata* aqueous extract at the doses of 50 and 200 mg/kg. MeOH + DMBA, Animals treated with the *F. umbellata* methanol extract at the dose of 50 mg/kg. TAM + DMBA, Animals treated with tamoxifen (3.3 mg/kg). All groups excepting the normal group (NOR) received an intragastric dose of DMBA at the dose of 50 mg/kg followed by a 10-day subcutaneous administration of 17β-estradiol (5 mg/kg). Data are represented as mean ± SEM (*n* = 10). * *p* < 0.05, ** *p* < 0.01 and *** *p* < 0.001 as compared to DMBA control.

**Table 6:** Effects of *F. umbellata* extracts on hematological parameters after 20 weeks of treatment.

Items	Control	DMBA	EA 50 + DMBA	EA 200 + DMBA	MeOH 50 + DMBA	Tamox + DMBA
WBC (×10^3^ µL^−1^)	1.91 ± 0.34	2.51 ± 0.40	3.9 ± 0.86	3.21 ± 0.67	2.3 ± 0.32	2.15 ± 0.22
Lymphocytes (%)	61.21 ± 4.88	59.48 ± 3.83	58.23 ± 3.62	62.15 ± 2.91	62.08 ± 3.04	58.55 ± 2.35
Monocytes (%)	6.65 ± 0.42	7.33 ± 0.60	6.61 ± 0.42	6.91 ± 0.81	7.28 ± 0.74	6.75 ± 0.52
Granulocytes (%)	32.13 ± 4.56	33.18 ± 3.68	35.15 ± 3.28	30.93 ± 2.28	30.63 ± 2.35	34.7 ± 1.86
RBC (×10^3^ µL^−1^)	7.22 ± 0.48	6.96 ± 0.40 ^#^	7.52 ± 0.23	7.60 ± 0.17	7.74 ± 0.18	7.28 ± 0.24
Hematocrit (%)	43.12 ± 3.43	42.16 ± 2.59	44.61 ± 1.39	45.1 ± 0.98	47.11 ± 1.17	43.73 ± 1.26
MCV (fL)	59.85 ± 1.12	60.51 ± 0.29	59.35 ± 0.54	59.38 ± 0.7	60.91 ± 0.39	60.16 ± 0.35
Platelets (×10^3^ µL^−1^)	378.2 ± 41.73	408.5 ± 18.75	422.83 ± 20.45	425.66 ± 32.12	455 ± 38.09	478.16 ± 15.03
MCH (pg)	18.68 ± 0.32	19.3 ± 0.25	18.65 ± 0.23	18.73 ± 0.20	18.96 ± 0.16	18.3 ± 0.15*
Hemoglobin (g/dL)	13.58 ± 1.08	13.51 ± 0.90	14.08 ± 0.52	14.28 ± 0.32	14.75 ± 0.42	13.41 ± 0.44
MCHC (g/dL)	31.31 ± 0.22	31.96 ± 0.28	31.48 ± 0.27	31.61 ± 0.43	31.23 ± 0.16	30.6 ± 0.22**

Control, Normal control treated with 2% ethanol; DMBA, DMBA control treated with 2% ethanol; EA + DMBA, Animals treated with the *F. umbellata* aqueous extract at the doses of 50 and 200 mg/kg. MeOH + DMBA, Animals treated with the *F. umbellata* methanol extract at the dose of 50 mg/kg. TAM + DMBA, Animals treated with tamoxifen (3.3 mg/kg). All groups excepting the normal group (NOR) received an intragastric dose of DMBA at the dose of 50 mg/kg followed by a 10-day subcutaneous administration of 17β-estradiol (5 mg/kg). Data are represented as mean ± SEM (*n* = 10). * *p* < 0.05 and ** *p* < 0.01 as compared to DMBA control; ^#^ *p* < 0.05 as compared to normal control.

## Supplementary Materials

Supplementary materials can be found at www.mdpi.com/1422-0067/18/6/1073/s1.

## Abbreviations

AE	Aqueous extract of *F. umbellata*
BW	Body weight
CC_50_	CC_50_, cytotoxic concentration for 50% of the cells
DCM	Dichloromethane
DMEM	Dulbecco’s Modified Eagle Medium
DMBA	7,12-Dimethylbenz(a)anththracene
DMSO	Dimethylsulfoxide
ER	Estrogen receptor
FBS	Fetal bovine serum
FCS	Fetal calf serum
HEPES	4-(2-Hydroxyethyl)-1-piperazineethanesulfonic acid
Hb	Hemoglobin
Ht	Hematocrit
MDA	Malondialdehyde
MCH	Mean corpuscular hemoglobin
MCHC	Mean corpuscular hemoglobin concentration
MCV	Mean corpuscular volume
MeOH	Methanol
NHC	National Herbarium of Cameroon
NOR	Normal control
RBC	Red blood cell
RPMI	Roswell Park Memorial Institute medium
ROS	Reactive oxygen species
SEM	Standard error of mean
TAM	Tamoxifen
