# Supplementary material for: Ficus umbellata Vahl. (Moraceae) Stem Bark Extracts Exert Antitumor Activities In Vitro and In Vivo
Source: Int J Mol Sci. 2017 May 23;18(6):1073. doi: 10.3390/ijms18061073 (PMC5485928; doi:10.3390/ijms18061073)
Supplement: Supplementary file 1 [file ijms-18-01073-s001.pdf]

Ficus umbellata Vahl. (Moraceae) Stem Bark Extracts Exert Antitumor Activities In vitro and In vivo

Kevine Kamga Silihe, Stéphane Zingue, Evelyn Winter, Charline Florence Awounfack, Anupam Bishayee, Nishil N. Desai, Leônidas João Mello Jr, Thomas Michel, Francine Nzufo Tankeu, Derek Tantoh Ndinteh, Sara Honorine Riwom, Dieudonné Njamen and Tânia Beatriz Creczynski-Pasa[4]

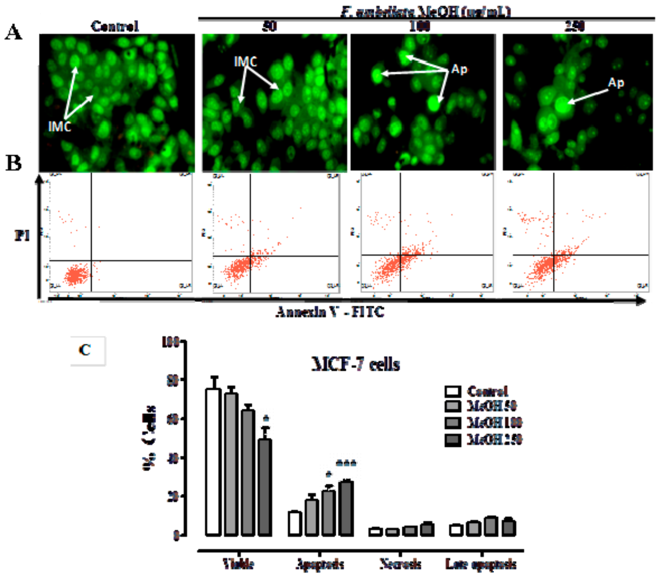

Figure S1. Cell death mechanism. Representative fluorescence microscopic images (400×) of cells double-stained with acridine orange (0.3 mg/mL) and ethidium bromide (1 mg/mL) (**A**); and dot plot representative of one experiment of apoptosis measurement by Annexin-V-FITC/PI staining (**B**). MCF-7 cells were treated for 24 h to *F. umbellata* extracts at concentrations of 45, 90 and 180 µg/mL. (**C**) Graph showing the mean±SEM of three independent experiments. $^{*}p < 0.05$, $^{***}p < 0.001$ as compared with control. IMC, intact membrane cell; Ap, apoptotic cells, Ne, necrotic cells.

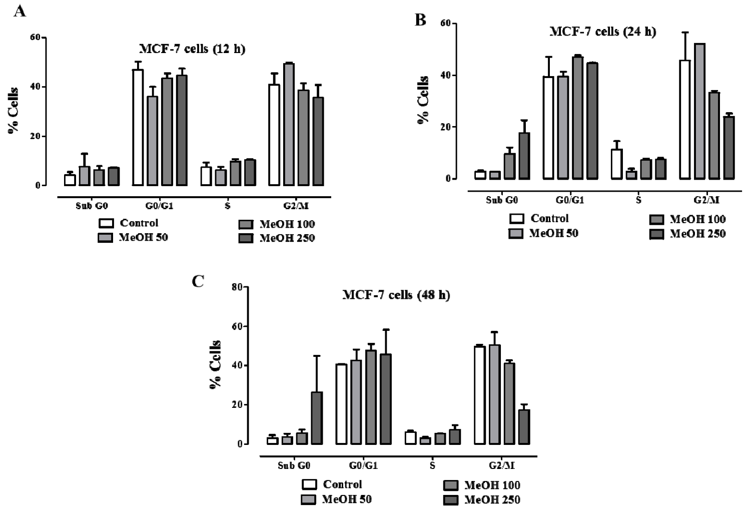

Figure S2. Cell cycle analysis. Effect of *F. umbellata* extracts on cell cycle distribution in MCF-7 cells after: 12 h (**A**); 24 h (**B**); and 48 h (**C**). Cells were treated for 24 h with 45, 90 and 180 µg/mL of extracts and staining with PI. Following flow cytometry, cellular DNA profile was analyzed using the software WinMDI 2.9. Data represent the percentage of cell counts in each cell cycle phase. The results are expressed as the mean±SEM of three independent experiments. No significant change was noted.
